# What are the influencing factors of online learning engagement? A systematic literature review

**DOI:** 10.3389/fpsyg.2025.1542652

**Published:** 2025-03-17

**Authors:** Juan Hu, Wen Xiao

**Affiliations:** ^1^School of Computer and Software, Anhui Institute of Information Technology, Wuhu, China; ^2^School of Educational Science, Anhui Normal University, Wuhu, China

**Keywords:** online learning, engagement, review, influencing factors, strategies

## Abstract

Since the onset of the COVID-19 pandemic in early 2020, online learning has gained widespread adoption as a learning mode in both K-12 and higher education. Learning engagement serves as a crucial indicator of learning quality and is highly correlated with students’ persistence, satisfaction, and academic performance. Numerous researchers have conducted investigations into the factors that influence online learning engagement. This study employs a systematic literature review methodology to synthesize 55 empirical studies published between January 2020 and July 2023. The research findings reveal the following: (1) Community of Inquiry Theory, Self-determination Theory, Social Cognition Theory, Transaction Distance Theory, and Technology Acceptance Model are the most frequently utilized theories employed by researchers to analyze the influencing factors of online learning engagement. (2) Factors that influence online learning engagement from the learners’ perspective include Motivation, Digital Experience and Literacy, Emotions and Regulatory Strategies, Psychology, Self-Perception, Self-efficacy, and Self-Directed Learning. Additionally, factors from the environment encompass Instrument, Task characteristics, Digital Platforms and Equipment, Physical Environment, Collaboration, and Interaction. (3) Effective strategies to enhance online learning engagement comprise setting clear learning goals for learners, improving their information and social media literacy, strengthening their self-directed learning ability, providing robust instructor support, and creating an optimal learning environment. Through this comprehensive review, researchers interested in this topic will gain a broader understanding, while also obtaining evidence-based insights and valuable recommendations for future research.

## 1 Introduction

Since the onset of the COVID-19 pandemic in early 2020, online learning has gained popularity as an alternative to traditional face-to-face learning in K-12 and higher education (Galea et al., 2020; Huang et al., 2020; Mailizar et al., 2020). Many educational institutions now provide learners with digital resources and learning activities through online learning platforms such as Learning Management Systems (LMS) and Massive Online Open Courses (MOOCs) to facilitate asynchronous and personalized learning (d’Orville, 2020; Sahu, 2020). However, recent studies have indicated that learners often feel distant and disconnected in online learning environments, which can negatively impact their performance and lead to high dropout rates (Donnelly and Patrinos, 2021; Hoi and Le Hang, 2021). Therefore, there is a pressing need to investigate the factors that influence online learning engagement and find ways to improve it.

## 2 Literature review

Learning engagement is a crucial indicator for measuring learners’ participation in online learning (Coates, 2010; Dixson, 2015; Luan et al., 2023), as it has a significant impact on learners’ satisfaction and academic performance (Chan et al., 2021; Kuh, 2010; Rita, 2011). The term “learning engagement” refers to the effort, time, and energy that students invest in academic experiences and educational activities with the aim of achieving desired learning outcomes (Astin, 2014). Its conceptual structure has been extensively described (Appleton et al., 2008; Hoi and Le Hang, 2021; Redmond et al., 2018; Reschly and Christenson, 2012), with the three-dimensional structure that includes cognitive, behavioral, and emotional engagement being the most frequently used by researchers (Fredricks et al., 2004; Kuh, 2009; Sun and Rueda, 2012). Wang et al. (2016) further added social engagement to the conceptual structure of learning engagement to emphasize the importance of learner interaction activities.

Due to the asynchronous and autonomous nature of online learning, learning engagement has gained increased importance. Several researchers have conducted comprehensive surveys about online learning engagement. Sima et al. summarized 19 indicators of learners’ behavioral engagement that could be extracted from LMS logs and demonstrated a significant relationship between learning engagement and academic performance (Caspari-Sadeghi, 2022). Golchehreh et al. reviewed 32 articles, resulting in 27 indicators that were categorized into three themes to measure the behavioral engagement of online learners (Ahmadi et al., 2023). Nurul et al. conducted a survey of 42 studies using the Preferred Reporting Items for Systematic Reviews and Meta-Analyses (PRISMA) methodology, summarizing the types of online learning engagement, the purpose of using learning analytics to analyze learning engagement, and its influence. Their findings indicate that learning analytics is an influential means of measuring and predicting online learning engagement, and there is a significant positive correlation between learning engagement and academic performance (Johar et al., 2023). Shofiyati et al. reviewed 47 studies on automatic recognition of learning engagement in the past 3 years and highlighted that researchers typically employ machine learning methods to automatically measure learners’ emotional engagement (Karimah and Hasegawa, 2022).

There are also some case studies on the influencing factors of online learning engagement. Elshami et al. identified the factors that affect student engagement in online learning in medical and health science colleges. The results showed high agreement between students and faculty on factors supporting online learning engagement, highlighting the importance of techno-pedagogical skills, self-directed learning, and the roles of peer-assisted and collaborative learning (Elshami et al., 2022). Werang and Leba (2022) explored online lecturers’ perceptions of factors affecting student engagement in online teaching and learning offered at Musamus University, Indonesia. Their study highlighted that limited access to technology, poor learning habits, and lack of digital skills are key factors affecting student engagement in online learning, emphasizing the need for improved resources and support. Abubakari, M.S. et al. conducted a study intending to model online learning engagement of international students studying in Indonesia to determine which factors affect learner engagement (Abubakari et al., 2022). The results showed that university support, motivation, and personal innovativeness were the significant predictors of international students’ engagement in online learning. Fan, Si et al. aimed to identify factors influencing student engagement in online and blended courses at one Australian regional university (Fan et al., 2021). They found a positive correlation between student engagement and teacher input, but a negative correlation with course content when it exceeded a certain threshold. However, these studies are mainly focus on students in specific disciplines or regions, and mainly use questionnaire surveys.

Despite these contributions, the current literature lacks a systematic review addressing the underlying factors that influence online learning engagement. Specifically, previous reviews, such as Rui et al.’s analysis of MOOCs, have highlighted the methods for measuring engagement but have not sufficiently explored the theories, models, or taxonomies explaining the influencing factors (Wang R. et al., 2022). The gap in understanding these factors, particularly in the context of recent developments in online learning, calls for a comprehensive synthesis of empirical research.

## 3 Methodology

To address the knowledge gap discussed earlier, we conducted a systematic literature review to explore and summarize empirical research on the influencing factors of online learning engagement published from January 2020 to July 2023. This review aims to provide a deeper understanding of the factors shaping engagement and how they are theorized and modeled in current research. Specifically, the study answers three key research questions:

RQ1. What theories or models have researchers employed to identify the influencing factors of online learning engagement?

RQ2. What is the taxonomy of the influencing factors of online learning engagement?

RQ3. How can learners’ online learning engagement be improved?

The Preferred Reporting Items for Systematic Reviews and Meta-Analysis (PRISMA) methodology used in this study is currently the most widely accepted approach for conducting literature reviews. It comprises four main steps: (1) determining inclusion and exclusion criteria for the literature, (2) devising search strategies, (3) retrieving and screening the identified literature, and (4) evaluating the selected literature to synthesize and analyze research findings. Our study followed these steps to ensure rigor in the systematic review process.

### 3.1 Inclusion and exclusion criteria

The inclusion criteria for literature selection in this study, based on the research questions, are outlined in Table 1.

**Table 1:** Inclusion criteria for selecting literature in this study.

Inclusion criteria	Description
Language of publication	English
Year of publication	From January 2020 to July 2023
Availability of full text	Full text is accessible for evaluate
Publication type	Article, not Letter, Editorial Material
Learning scenarios	Online Learning, rather than face to face or blending learning
Subject	The influencing factors of learning engagement, rather than the conceptual structure or measurement of learning engagement
Research type	Empirical research with hypotheses and data-based testing or Qualitative research with interview and content analysis

This review excludes literature written in languages other than English and those published outside the specified time frame. Furthermore, it specifically focuses on the influencing factors of online learning engagement, thus excluding studies that do not pertain to online learning scenarios. As mentioned in the previous section, researchers commonly adopt the conceptual structure of learning engagement, which encompasses cognitive, behavioral, emotional, and social dimensions (Wang et al., 2016). In line with this established framework, our study also utilized this conceptual structure. However, given the specific focus on investigating the influencing factors of online learning engagement, we excluded literature pertaining solely to the conceptual structure and measurement of online learning engagement. Lastly, we excluded studies that did not employ data or interviews to validate research hypotheses, such as summary or speculative research.

### 3.2 Search and filtering

**Figure 1:**
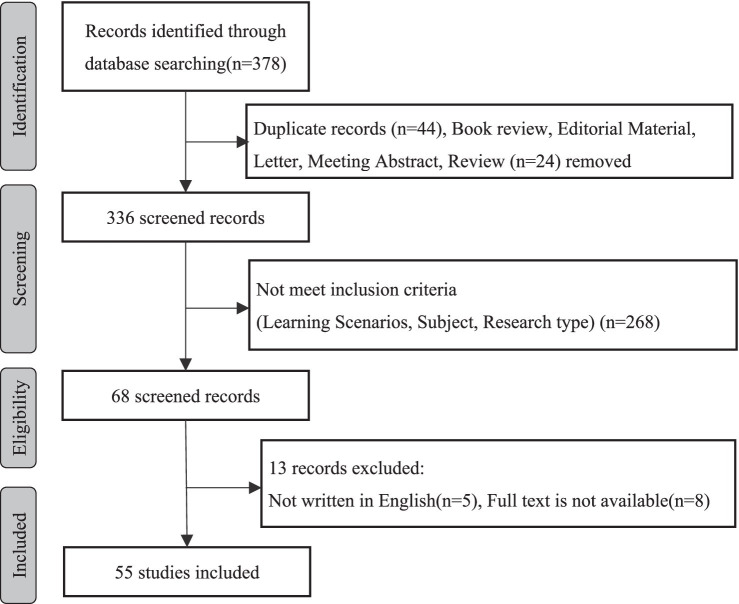
Methodology of literature search and filter in this review.

To ensure the inclusion of literature directly relevant to our research question, we utilized four databases as data sources: Web of Science, Science Direct, Springer Link, and Wiley. Given that the majority of literature pertaining to our research topic originates from social sciences disciplines such as education and psychology, we deliberately excluded databases known for engineering and medical literature, such as IEEE and PubMed. For our search, we employed the search terms “online learning engagement” and “MOOC student engagement” using the following query: ((TITLE-ABS-KEY (“Learning Engagement”) OR TITLE-ABS-KEY (“Student Engagement”)) AND (TITLE-ABS-KEY (“online”) OR TITLE-ABS-KEY (“MOOC”))). As shown in Figure 1, this search yielded a total of 378 articles during this stage, based on the Year of Publication.

To avoid duplication, we identified and removed duplicate literature (*n* = 44) that appeared across different databases. Furthermore, we excluded any literature that did not conform to the selection criteria outlined in Table 1, excluding an additional 268 articles. The remaining 336 articles were subjected to careful scrutiny, and after examining the full text of 68 articles, we further excluded 13 articles that did not meet our inclusion criteria.

The methodology adopted for conducting the literature search and filtering process is outlined in Figure 1.

## 4 Descriptive analysis

We conducted a frequency-based descriptive statistical analysis of the research results from the selected papers and performed comparisons and discussions. The results are as follows.

### 4.1 Source of paper

**Figure 2:**
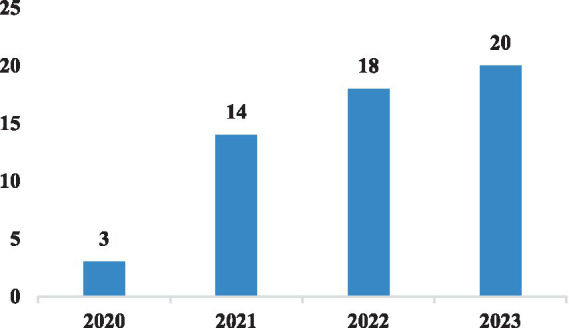
The publication years of the selected papers.

In the wake of the COVID-19 pandemic that emerged in early 2020, online learning has emerged as the primary alternative to traditional face-to-face teaching. Figure 2 illustrates the publication years of the 55 selected papers included in this review. It is evident that online learning engagement has garnered increased attention from researchers following the pandemic, resulting in a significant rise in the number of papers published after 2020. These selected papers were published across a total of 35 journals, with notable contributions from journals such as SUSTAINABILITY (*n* = 6), Computers & EDUCATION (*n* = 4), FRONTIERS IN PSYCHOLOGY (*n* = 4), JOURNAL OF COMPUTER ASSISTED LEARNING (*n* = 4), and INTERACTIVE LEARNING ENVIRONMENTS (*n* = 3), with each having published over three papers. The majority of these journals fall within the domains of pedagogy and psychology, particularly those with a primary focus on leveraging digital technology to enhance education.

### 4.2 Participants in the selected paper

**Figure 3:**
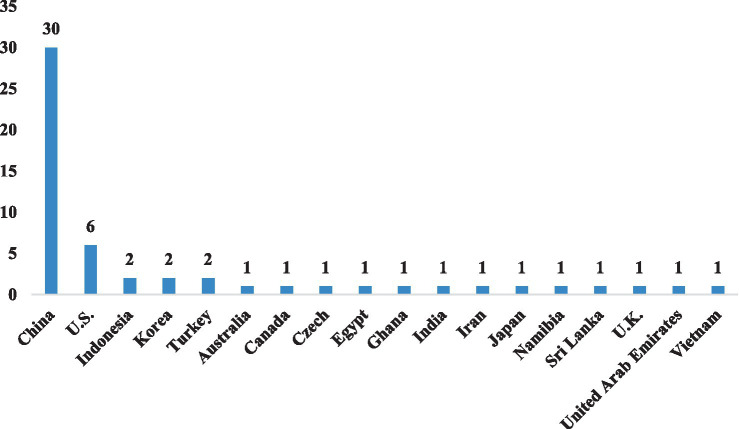
The countries in which the selected studies were conducted.

The COVID-19 pandemic has had a profound impact on the world, leading to the closure of schools in almost all countries over a period of time and prompting a rapid shift toward online learning. Consequently, there has been a growing interest in identifying the factors that influence online learning engagement across many countries worldwide. As Figure 3 illustrates, the 55 selected studies in this review were conducted in 18 countries, with China (*n* = 30) and the United States (*n* = 6) ranking first and second, respectively. Given that China boasts the largest education system globally, characterized by significant regional differences, and that Chinese schools were among the first to be affected by the pandemic (Chen et al., 2020a; Chen et al., 2020b), this may explain why the majority of studies originated from China.

**Figure 4:**
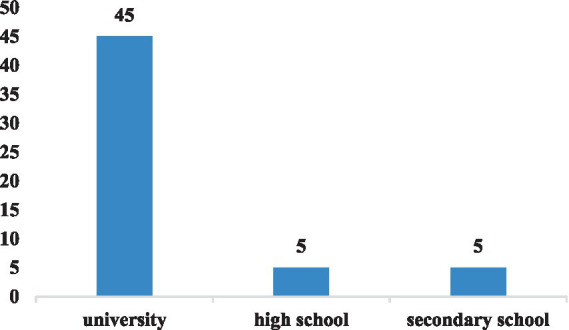
Types of learners in the selected papers.

From the perspective of learners’ types (as shown in Figure 4), the majority of research on online learning engagement has focused on college students (*n* = 45), while comparatively little attention has been paid to middle school (*n* = 5) and high school students (*n* = 5). This can be attributed to the fact that college students tend to have better self-directed learning abilities and universities often provide necessary online learning environments, such as digital platforms and networks, which facilitated greater engagement in online learning during the pandemic. In contrast, K-12 students may have faced greater challenges given their comparatively less-developed independent learning skills and the need for parental support (Neuwirth et al., 2021; Rashid and Yadav, 2020).

### 4.3 Dimensions of online learning engagement

**Figure 5:**
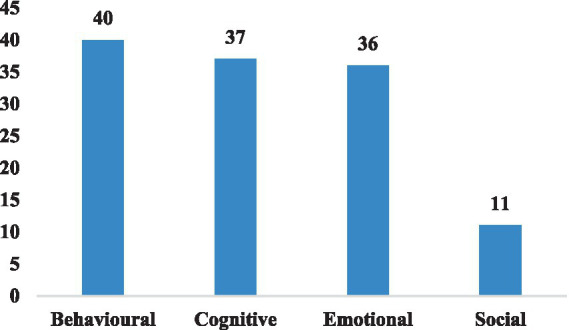
The number of different dimensions of online learning engagement in selected papers.

As Figure 5 illustrates, among the selected studies, 12 did not explicitly indicate the dimensions of online learning engagement, while the majority of researchers employed a conceptual framework comprising of behavioral, cognitive, and emotional engagement (*n* = 23). Additionally, many researchers used the four-dimensional conceptual framework revised by Fredricks et al. (*n* = 11). Some studies integrated both behavioral and emotional engagement (*n* = 3), while others focused solely on students’ online behavioral engagement (*n* = 4) or cognitive engagement (*n* = 2). Notably, social engagement was the least explored dimension in the reviewed literatures.

### 4.4 Methodology of data collection and analysis

Regarding data collection methods, the majority of researchers (*n* = 42) utilize questionnaires as a means to collect empirical data. Additionally, a considerable number of researchers employ semi-structured interviews to gather responses from students and teachers regarding specific questions (*n* = 7). Some researchers also analyze demographic features of learners, system logs, online discussion texts, and other data stored on online learning platforms to examine behavior and social engagement (*n* = 5). Moreover, there have been instances where researchers have utilized neurophysiological instruments to collect students’ EEG (Electroencephalogram) records in order to investigate cognitive engagement in online learning (*n* = 1).

**Figure 6:**
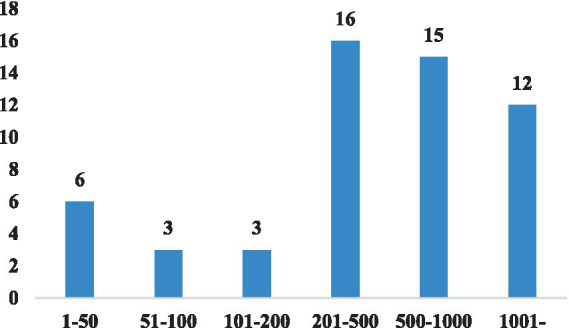
The number of samples in selected papers.

Furthermore, Figure 6 presents the distribution of sample sizes in the collected data. Typically, studies utilizing neurophysiological instruments and interviews tend to have relatively small sample sizes (ranging from 1 to 50). On the other hand, the largest sample size of 5,906 is derived from logs obtained from the learning management system. It is worth noting that the majority of studies have sample sizes exceeding 200, which can be considered as large-sample sizes in statistical analysis.

Regarding data analysis methods, researchers commonly employ Confirmatory Factor Analysis (CFA) to assess the reliability and validity of data collected through questionnaires. Structural Equation Modeling (SEM) is then utilized to validate hypotheses and examine the relationships between influencing factors and online learning engagement (*n* = 40). Additionally, correlation coefficients such as Pearson and Spearman are employed to indicate the relationship between influencing factors and online learning engagement (*n* = 11). Researchers often employ Analysis of Variance (ANOVA) and T-test to investigate the influence of different factors on students’ online learning engagement (*n* = 8). Regression analysis is also used by some researchers, as the coefficients in the regression equation can indicate the importance of different factors in relation to online learning engagement (*n* = 5). The mediating effects among influencing factors are typically tested using bootstrap tests (*n* = 11). For data collected through interviews, researchers commonly utilize topic analysis to extract key theme-words that identify the influencing factors of online learning engagement (*n* = 7). Moreover, text mining and Epistemic Network Analysis are occasionally employed to analyze online discussion texts (*n* = 1).

## 5 Results

In this section, we synthesize the key findings of this study by reviewing three research questions.

RQ1. What theories or models have researchers employed to identify the influencing factors of online learning engagement?

**Figure 7:**
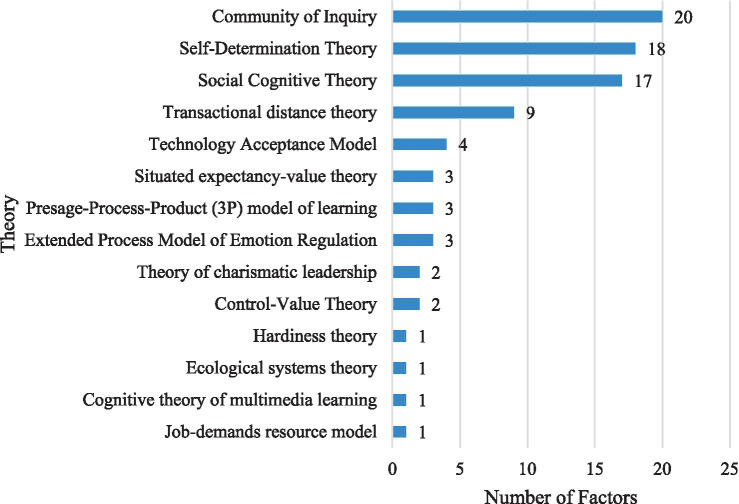
The theories explicitly indicated by the researcher in the selected papers.

Researchers typically draw on theories from various fields, including psychology, sociology, and management, to put forth hypotheses concerning the factors that influence online learning engagement. These hypotheses are subsequently tested using collected data or interview answers. In the selected papers, Figure 7 highlights the theories explicitly indicated by the researchers.

### 5.1 Community of Inquiry Theory

The Community of Inquiry (COI) theory has emerged as the most widely utilized theory among researchers due to its focus on comprehending social interaction and cognitive processes in online learning and distance education (Garrison, 2016). This theory posits that in an online learning environment, learners can form a community that enhances learning efficiency through collaborative exploration, cooperation, and discussion. The COI theory encompasses three fundamental factors: Cognitive Presence, Social Presence, and Teaching Presence. These factors encompass learners’ cognitive activities and knowledge construction during exploratory learning, social interactions and emotional connections between learners, as well as teachers’ roles and actions in online learning. Strengthening these three presence factors can facilitate the formation and development of learning communities.

Teaching Presence is considered the most influential factor, and multiple research studies (*n* = 14) have demonstrated that teachers’ design and organization of course, facilitation of discussions, direct instruction, assessment and feedback, technical support, and other behaviors can significantly impact students’ engagement in online learning. This finding aligns with observations made in various other learning environments (Bond, 2020; Bryson and Hand, 2007; Hew, 2016).

One prominent characteristic of online learning is the temporal and spatial separation between students and teachers, which presents challenges for interaction (Watts, 2016). Social Presence, encompassing aspects such as social respect, social sharing, open-mindedness, social identity, and intimacy (Sung and Mayer, 2012), can help alleviate feelings of loneliness and anxiety among students in online learning, thus enhancing their level of engagement (Cobb, 2009).

Moreover, findings from a single study indicate that learners’ cognitive presence, reflecting their knowledge absorption and construction, also significantly influences their engagement in online learning.

### 5.2 Self-determination theory

The self-determination theory (SDT) (Deci and Ryan, 2013) is one of the most widely recognized theories in the field of pedagogy regarding academic motivation (Ryan and Deci, 2017, 2020). According to this theory, the core factors of autonomy, competence, and relatedness are fundamental needs that can enhance learners’ internal motivation and engagement (Black and Deci, 2000). Numerous studies have highlighted the significant influence of learners’ motivation and satisfaction of these three fundamental needs on their engagement in online learning.

Research findings indicate that both internal motivation, derived from learners’ intrinsic interest, curiosity, autonomy, and the pursuit of task value and enjoyment, as well as external motivation driven by external incentives such as rewards, punishments, evaluations, and recognition, significantly impact online learning engagement (Zhou et al., 2022; Chen, 2023). These findings align with observations from face-to-face and blended learning contexts (Buil et al., 2020; Dincer et al., 2019; Lietaert et al., 2015; Vollet et al., 2017).

Among the three fundamental needs, perceived autonomy has received the most attention from researchers (*n* = 6). Students with a stronger sense of autonomy and intrinsic motivation are more likely to actively participate in online learning activities and tasks. Similarly, learners with a stronger sense of relatedness can overcome feelings of isolation and alienation in online learning (*n* = 3), as they maintain closer relationships with teachers, resulting in higher levels of engagement. Perceived competence, which refers to learners’ subjective perception and evaluation of their ability to complete online courses or tasks, exhibits a positive correlation with academic motivation and engagement (*n* = 3).

In addition to exploring the three fundamental needs of learners, researchers have also investigated how these needs can be met in online learning environments. The selected paper’s findings indicate that teacher support plays a crucial role in meeting learners’ needs. This support includes providing and recommending various types of digital resources to support students’ learning anytime and anywhere, offering clear guidance on digital submission and technical issues, utilizing carefully designed learning materials, providing multimodal feedback to students in asynchronous forums, hosting real-time interactive courses through instant messaging software, and using visual aids such as images and emoticons to facilitate communication and create a positive mood. These findings are consistent with those from traditional learning scenarios, and there is a significant positive correlation between teacher support and online learning engagement (Chiu, 2022; Li et al., 2022).

Secondly, a supportive online learning environment is conducive to meeting the needs of learners, particularly with easy-to-use online learning platforms and sufficient home equipment and resources (Zang et al., 2022). Thirdly, learners’ self-regulated learning ability, digital literacy, and prior learning experience are also critical factors in meeting their fundamental needs (Chiu, 2021), which is consistent with the conclusions of other studies (Hsu et al., 2019; Rosli et al., 2022).

### 5.3 Social Cognitive Theory

Social Cognitive Theory (SCT), also known as Social Learning Theory, emphasizes the interaction between cognition, behavior, and the environment in the process of individual learning and development. One of the core components of SCT is self-efficacy (Bandura, 1977). Self-efficacy refers to an individual’s confidence and judgment in their ability to successfully complete tasks, which subsequently influences their decision-making, effort, and persistence. In the context of online learning, self-efficacy is commonly referred to as academic self-efficacy. It pertains to learners’ self-perceived ability to accomplish various online learning activities, access resources, complete courses, and achieve desired academic grades (Artino and McCoach, 2008; Cheng and Tsai, 2011; Cho et al., 2017; Zimmerman and Kulikowich, 2016). At the same time, individuals can adjust themselves through observation and reflection, in order to rise to challenges from the external environment. Based on this theory, many researchers in the selected papers have verified that self-efficacy and self-regulation are significant influencing factors for online learning engagement. In online learning, learners’ self-efficacy is self-assessment to complete an online course, use tools in a Course Management System, interact with instructors in an online course, interact with classmates for academic purposes (Derakhshan and Fathi, 2023).

In addition, researchers have also identified ICT self-efficacy, which encompasses the sense of self-competence (e.g., one’s perception of their own online learning goals), the sense of self-effort (e.g., one’s ability to concentrate on online learning), and the sense of environmental control (e.g., one’s feelings about the online learning environment) (Feng et al., 2023). Other factors such as technology use, time management, and the online learning environment have also been found to influence learners’ self-efficacy in online learning (Heo et al., 2021). Learners’ self-efficacy in online learning is centered around their self-assessment of their ability to utilize technology effectively and adapt to technological learning environments.

Self-regulated learning is another crucial component of social cognitive theory (Garrison, 1997; Kicken et al., 2009). This theory suggests that learners can improve their performance by independently setting goals, monitoring their progress, adjusting learning strategies, and evaluating their outcomes (Zimmerman, 2002; Zimmerman, 2002). Previous research has shown that learners’ self-regulated learning skills and abilities have a significant impact on learning engagement (Stefansson et al., 2018), particularly in online learning environments (Coelho et al., 2019; Doo et al., 2020; Findlater et al., 2012). Among the six selected papers, students’ self-regulated learning abilities are composed of six aspects: goal setting, time management, environmental construction, task strategy, seeking help, and self-evaluation (Barnard et al., 2009). These aspects highlight the importance of learners taking control of their learning processes and being proactive in achieving their goals.

### 5.4 Transactional Distance Theory

Transactional Distance Theory (TDT) is a theoretical framework that illustrates how psychological and communicative distance between learners and instructors can impact learning outcomes (Moore, 1993). The term “transactional distance” refers to the gap in communication and understanding caused by physical distance between learners and instructors, which has been found to be closely related to learning engagement in numerous previous studies (Arbaugh and Rau, 2010; Martin and Borup, 2022; Piccoli and Ives, 2001).

In online learning, where learners and instructors are separated in time and space, positive interaction plays a crucial role in reducing potential misunderstandings caused by psychological and communicative distance. Enhancing online interaction is the best way to motivate learners, stimulate their enthusiasm for learning, and improve their learning efficiency (Chen C. M. et al., 2022; Lai et al., 2019; Wang et al., 2022). This highlights the importance of creating opportunities for communication and collaboration in online learning environments to decrease transactional distance and enhance learning engagement.

Interaction in the context of online learning can be categorized into three types (Moore, 1993): (1) Learner-Instructor interaction refers to bi-directional communication between students and teachers. It involves activities such as asking questions, seeking support, and receiving encouragement from instructors; (2) Learner-Learner interaction involves communication and collaboration among individual learners or in groups. Learners engage in activities such as exchanging ideas, discussing course-related topics, and providing feedback to their peers. Learner-learner interaction promotes social learning, peer support, and the sharing of diverse perspectives, which can enhance learning engagement; (3) Learner-Content interaction focuses on learners actively engaging with the course content, constructing meaning, and solving problems. It includes activities such as reading course materials, completing assignments, and participating in simulations or online activities, allowing learners to make connections, apply knowledge, and develop a deeper understanding of the subject matter.

The selected papers provide evidence that these three types of online interactions have a significant influence on online learning engagement (Chen, 2023; Wang et al., 2022; Guo et al., 2023). Additionally, the overall level of online interaction has also been found to have a significant impact on learning engagement (Gherghel et al., 2023; Miao and Ma, 2022; Tsai et al., 2021). These findings emphasize the importance of fostering meaningful interactions in online learning environments to promote learner engagement and improve learning outcomes.

### 5.5 Technology Acceptance Model

The Technology Acceptance Model (TAM) is a theoretical model widely used to explain and predict individuals’ willingness to adopt and use new technologies, particularly in online environments that rely on information and communication technology support (Davis, 1989). TAM suggests that the adoption of a new technology is primarily influenced by two core factors: Perceived Usefulness and Perceived Ease of Use.

Perceived Usefulness refers to learners’ subjective evaluation of the benefits and contributions of learning support systems in accomplishing their learning tasks. It relates to how learners perceive that the technology can enhance their learning experience, improve their performance, or help them achieve their learning objectives. Perceived Ease of Use, on the other hand, refers to learners’ subjective evaluation of the difficulty associated with using online learning systems. It reflects learners’ perception of how easy or convenient it is to interact with the technology, navigate through the learning materials, and perform various tasks within the online learning environment.

Previous studies have consistently demonstrated that learners’ perceptions of the usefulness and ease of use of learning support systems significantly impact their learning engagement (Kuzu and Gunuc, 2015; O’Shea et al., 2014; Waite et al., 2013). Specifically, when learners perceive a technology as useful and easy to use, they are more likely to engage actively in the learning process and exhibit higher levels of motivation and satisfaction.

The selected papers you mentioned also validate the influence of perceived usefulness and perceived ease of use on online learning engagement (El-Sayad et al., 2021; Jung and Lee, 2018; Ma et al., 2022). These findings highlight the importance of designing online learning systems that are perceived as useful and user-friendly to promote learner engagement and adoption of the technology.

### 5.6 Other theories and models

In addition to the five common theories, Figure 7 highlights the use of theories from emotional regulation, sociology, and management to analyze factors influencing online learning engagement.

The Extended Process Model of Emotion Regulation (EPMER) (Gross, 1998) highlights the significant relationship between emotional regulation and individual goal achievement. Two common emotion regulation strategies, namely Reappraisal and Suppression, have been found to influence positive and negative academic emotions (Naragon-Gainey et al., 2017), which in turn affect learning engagement (Riegel and Evans, 2021). Effective regulation of emotions results in a more constructive online learning engagement (Bakır-Yalçın and Usluel, 2023).

In the Presage-Process-Product (3P) model of learning, positive or negative academic emotions are classified as part of the “Process” component (Biggs, 1993). In the context of online learning, the “Presage” component encompasses information literacy, while the “Product” component refers to online learning engagement (Li et al., 2023). Various studies have shown that learners’ information literacy significantly influences their online learning engagement (Avcı and Ergün, 2022; Bergdahl et al., 2020; Fosnacht, 2020).

Both the Situated Expectancy Value Theory and the Control Value Theory center around individuals’ expectations and values regarding learning or behavior, highlighting the impact of environments on shaping these expectations and values. Consequently, the Perceived Value of Learning Goals (Sun et al., 2023) and Task Value (Bakır-Yalçın and Usluel, 2023) have been confirmed to exert a substantial influence on online learning engagement.

According to the Theory of Charismatic Leadership (House, 1976), charismatic leaders can leave a positive impression on individuals, establish high expectations for their followers, stimulate enthusiasm, and enhance productivity (Miner, 2005). Instructors who possess charisma and leadership skills can utilize inspiring and engaging speech, as well as effectively leverage technology to provide support and resources for the benefit of students (Queen, 2022). Consequently, the charismatic leadership skills and technology use skills of instructors have also been shown to significantly influence online learning engagement (Hazzam and Wilkins, 2023).

The Job Demands Resource (JDR) model (Bakker and Demerouti, 2007) suggests that individuals may experience job burnout when their needs are not met by available resources (Demerouti et al., 2001). Furthermore, perceived social support has been shown to significantly influence learners’ engagement t (Huang et al., 2024). Similarly, drawing from the ecological system theory (Bronfenbrenner, 1986), learning engagement is considered to be influenced by the interaction of individual and situational factors within an ecosystem (Liu et al., 2018; Roffeei et al., 2015). Social support, specifically academic and emotional support provided by teachers and peers, has been identified as a significant influencing factor for learning engagement (Luan et al., 2023).

Finally, the visual and linguistic aspects of online learning content, especially when presented in video format, have been found to facilitate effective learning (Guo et al., 2014). Additionally, the social cues, such as the instructor’s voice and facial expressions, can enhance students’ engagement (Kizilcec et al., 2015; Korving et al., 2016). Consequently, researchers have employed the cognitive theory of multimedia learning (Mayer, 2005) to investigate the factors influencing online learning engagement. Some research findings suggest that videos providing more visual cues and richer vocabulary information have a significantly positive impact on online learning engagement (Lackmann et al., 2021).

These theories mentioned share several overlapping constructs that can help explain the influencing factors of online learning engagement. CoI highlights the importance of cognitive, social, and teaching presence, which aligns with SDT’s focus on intrinsic motivation and autonomy in learning. Both theories suggest that learners engage more deeply when they feel supported and have a sense of community. SCT complements this by focusing on self-efficacy and self-regulation, underscoring the role of learner motivation and the ability to manage one’s learning environment, which also resonates with TDT’s idea of reducing transactional distance. TDT proposes that engagement is hindered by perceived gaps in interaction between learners and instructors, while SCT emphasizes the role of social and cognitive interaction in overcoming this distance. TAM, which addresses technology acceptance, overlaps with CoI by stressing the importance of technology in facilitating meaningful interaction and engagement. Collectively, these theories highlight the importance of learner motivation, autonomy, self-regulation, social interaction, and the use of technology, suggesting that a combined framework can offer a comprehensive explanation of the factors driving online learning engagement.

RQ2. What is the taxonomy of the influencing factors of online learning engagement?

In addition to the classic theoretical models, numerous researchers have also explored potential factors that can influence online learning engagement. These factors have been derived from previous literature or practical experience. After conducting a comprehensive literature review, we classified the influencing factors used by researchers with similar connotations but described using different terms. To provide a clear framework, we proposed a binary taxonomy consisting of two main categories: learners and learning environments. The complete classification is presented in Tables 2, 3.

**Table 2:** Influencing factors from learners.

Type	Factors	Number of occurrences in the selected paper
Motivation	Internal	5
	External	
Experience and literacy	Prior knowledge	1
	Learning habits	1
	Digital nativity	1
	Information literacy	1
	Technology experience	1
	Social media literacy	1
Emotions and regulatory strategies	Academic emotions	8
	Regulation strategies	2
Demography	District	1
Psychology	Grit	1
	Academic hardiness	1
	Psychological safety	1
	Subjective wellbeing	1
Self-perception	Perceived educational situation	1
	Perceived autonomy	6
	Perceived competence	3
	Perceived relatedness	3
	Perceived value of knowing learning goals	1
	Perceived academic control	1
	perceived usefulness	1
	perceived ease of use	3
	perceived social support	1
Self-efficacy	Online course completion	13
	Peer interaction	
	Teacher-student interaction	
	Self-regulation	
	Learning management system	
	Time management	
	Technology using	
Self-directed learning	Self-directed learning	6
	Self-directed learning approach	1
	Self-directed learning attitude	1
	Self-directed learning skills	1

**Table 3:** Influencing factors from learning environment.

Type	Factors	Number of occurrences in the selected paper
Instructor	Teaching motivation	1
	Coaching and mentoring	1
	Humor	2
	Charismatic leadership skills	1
	Techno-pedagogical skills	2
	Teaching presence	14
Task	Task value	1
	Video format	1
Digital platforms and equipment	Online learning platforms	1
	Technical equipment	5
Physical environment	Ambient attributes	2
	Spatial attributes	
Collaboration and interaction	Collaborative learning	1
	Social presence	7
	Interactions	8

Based on our analysis of the selected papers, we categorized the 35 influencing factors from learners into eight distinct categories, which are presented in Table 2.

Firstly, internal motivation, stemming from factors such as learners’ interest, curiosity, autonomy, and sense of achievement, along with external motivation, derived from rewards, punishments, evaluations, and expectations, exert a substantial influence on online learning engagement. Among these, internal motivation serves as a persistent and long-term driving force for online learners, playing a more prominent role (Prakasha et al., 2023; Zang et al., 2022).

Secondly, given that online learning typically takes place within a technologically supported environment, the proficient use of online learning platforms, access to digital resources, and active participation in online learning activities are prerequisites for effective learning. Hence, technical factors like Digital Nativity and information literacy also hold significant sway over online learning engagement.

Thirdly, Happiness, hope, pride, and other positive academic emotions constitute the core of emotional engagement, playing pivotal roles in both cognitive and behavioral engagement. Conversely, negative emotions like boredom, anxiety, and depression can impede online learning engagement. Reappraisal and suppression are two emotional regulation strategies that, respectively, yield positive and negative academic emotions, thereby affecting online learning engagement (Zhoc et al., 2022).

Fourthly, the urban–rural divide can significantly impact students’ online learning engagement, likely due to the disparities in network infrastructure, equipment support, and teacher support between these regions (Zheng et al., 2023).

Finally, learners who possess characteristics such as perseverance, a sense of security, and subjective well-being tend to exhibit higher levels of learning engagement. The self-efficacy of learners is highly contextualized, and the seven factors of self-efficacy reflect the technical aspects of online learning. Given that instructors and learners in online learning are typically separated by time and space, learners’ attitudes, approaches, and self-directed learning skills also exert a substantial influence on their engagement.

Similarly, we have divided the 15 influencing factors from the learning environment into five distinct categories. These categories are outlined in Table 3, providing a comprehensive overview of the various aspects that impact online learning engagement.

According to Table 3, the most significant types of influencing factors in the learning environment are instructors, collaboration, and interaction. Despite the physical separation between instructors and students in online learning, their support, guidance, and supervision still play a vital role in enhancing learning engagement. Teaching presence encompasses various aspects such as setting clear learning objectives, designing engaging and adaptable learning activities that cater to students’ needs, selecting appropriate digital resources, and planning the learning process in advance.

In synchronous online learning, instructors directly impart knowledge and skills to students through explanations, demonstrations, and other approaches. They also provide timely feedback and evaluation to students. Additionally, teachers facilitate cooperation and mutual assistance among students by posing questions and encouraging critical thinking. In a technically-oriented online learning environment, instructors’ techno-pedagogical skills significantly influence learners’ engagement. These skills involve effectively utilizing technology to facilitate learning and create an optimal learning experience for students.

Social presence enables learners to connect with peers and instructors in a supportive and engaging environment. It facilitates the development of a sense of community, which in turn enhances learners’ emotional engagement and overall satisfaction. Interaction, including student–student, student-teacher, and student-content interactions, provides multiple opportunities for learning and problem-solving. Active participation in these interactions also promotes learners’ self-efficacy and self-directed learning skills, further enhancing their engagement in the online learning process.

RQ3. How can learners’ online learning engagement be improved?

In the selected papers, researchers have proposed strategies to purposefully improve learners’ online learning engagement based on the identified influencing factors. These strategies can be summarized into the following three aspects:

Firstly, key factors that influence online learning engagement include learners’ motivation, digital literacy, positive academic emotions, and self-directed learning abilities. To foster engagement, it is essential to set clear academic goals that help learners understand the value of online learning for personal and career development. Additionally, implementing incentives and penalties within the online learning environment can further drive engagement. Educational institutions should prioritize improving learners’ information and media literacy, enabling them to actively participate in technical aspects of online learning. Creating a supportive emotional atmosphere and guiding learners in managing their well-being through self-reflection is also vital. Cultivating self-management skills is crucial for enhancing self-regulated learning.

Secondly, instructor support plays a pivotal role in fostering engagement. Instructors should set clear academic expectations, continually improve their technical skills, and use various online tools to create interactive, supportive learning environments. Activities like group projects, case studies, online assessments, and peer support are valuable. Encouraging collaboration and active participation in discussions, question-asking, and sharing experiences can further enhance engagement. Personalized feedback and guidance help create a friendly learning environment, and instructors should also empower learners with autonomy in managing their learning progress.

Thirdly, the learning environment itself is another key factor. Institutions and families should create quiet, organized spaces, while online platforms should be user-friendly and stable. Governments can reduce the urban–rural education gap by providing necessary equipment and ensuring reliable internet access for all learners.

Considering the differences between secondary school and university students, different strategies should be adopted to enhance engagement. For secondary school students, engagement can be increased through more structured guidance and support. Teachers should provide clear instructions and frequent feedback to help students stay on track. Interactive activities such as gamified learning, quizzes, and group projects can foster collaboration and make learning more engaging. Since middle school students often need more supervision, regular check-ins and motivational rewards (such as badges or certificates) can encourage continued participation. Additionally, creating a positive emotional atmosphere and offering personal encouragement can boost their motivation. For university students, a greater emphasis should be placed on fostering independence and self-directed learning. While instructors should still offer guidance, university students benefit from having more autonomy in managing their learning. Setting challenging academic goals and offering opportunities for critical thinking, such as research projects, case studies, and peer discussions, can stimulate deeper engagement. Encouraging students to apply theoretical knowledge to real-world situations enhances their learning experience. In addition, providing platforms for networking and professional development, such as virtual seminars or industry-related discussions, can also increase motivation, helping students see the value of online learning for their career goals.

## 6 Summary and prospect

This study employed a systematic literature review approach to identify and analyze empirical studies on the influencing factors of online learning engagement following the onset of the COVID-19 pandemic in early 2020. A total of 55 relevant studies were selected and comprehensively examined from three main perspectives. The key findings are outlined below:

1. Since the COVID-19 pandemic, online learning has become the dominant educational method in both secondary and higher education. A large body of research has focused on online learning engagement, particularly among university students, with most studies coming from China and the United States. These studies often use questionnaires to gather data, and structural equation modeling is frequently employed for analysis.
2. Researchers tend to base their hypotheses on social and cognitive psychology theories to explore the factors that influence online learning engagement. Some of the most commonly used theories include the Community of Inquiry Theory, Self-Determination Theory, Social Cognitive Theory, Transactional Distance Theory, and the Technology Acceptance Model. These frameworks have been refined and adapted to better fit the technical aspects of online learning. Many studies integrate multiple theories to identify a range of factors that impact engagement.
3. The factors influencing online learning engagement are typically divided into two categories: learner-related and environmental factors. Learner-related factors include motivation, academic emotions, self-awareness, self-efficacy, and self-regulated learning abilities. Environmental factors, on the other hand, encompass the role of instructors, the quality of digital platforms and equipment, and the importance of collaboration and interaction within the online learning environment. These elements are considered crucial in shaping students’ engagement with online learning.

Based on the results of this review, our research recommendations for future research are as follows.

1. Firstly, it is crucial for researchers to focus more on learners in elementary and pre-elementary education, as well as those in continuing education and vocational training. The studies reviewed predominantly feature college students, with 81% of the participants from this group. There is a noticeable lack of representation from other educational stages, such as elementary, pre-elementary, and vocational education. It is worth noting that elementary and pre-elementary students and adult learners differ significantly from college students, and their online learning engagement may be influenced by distinct factors. Therefore, further research is needed to identify and understand the specific influencing factors that impact the online learning engagement of these learner groups.
2. Secondly, it is important to identify the influencing factors of online learning engagement based on multimodal data available on online learning platforms. In the selected papers, 95% of the empirical research studies used classical questionnaires and interviews to collect data, which were then analyzed and validated using statistical methods such as SEM, correlation, ANOVA, regression, etc. While questionnaires and structured interviews can effectively measure learners’ impersonal situations and subjective attitudes, they are subjective, time-consuming, and labor-intensive. On the other hand, multimodal data such as meta-attributes of learning resources and activities, logs of learning activities, learning community interactions, and demographic characteristics of teachers and students stored in online learning platforms can be analyzed through multimodal learning analytics investigate the influencing factors of online learning engagement. This approach can provide more objective, comprehensive, and accurate insights into learners’ engagement levels and the factors that affect them. Therefore, future research should employ a more diverse range of data collection methods to analyze the influencing factors of online learning engagement.
3. Thirdly, it is crucial for researchers to empirically validate intervention and enhancement strategies aimed at improving online learning engagement. While the selected papers have investigated and discussed the influencing factors of online learning engagement, and proposed various strategies to enhance it, there is a lack of subsequent empirical verification of these strategies.

Empirical validation can provide evidence to support the identified influencing factors and demonstrate their positive impact on online learning engagement. By conducting rigorous research, researchers can ensure that the strategies they recommend are accurate, reliable, and beneficial for learners. Various quantitative and qualitative research methods can be employed to analyze the data and determine the effectiveness of the strategies. Additionally, longitudinal studies can be conducted to examine the long-term impact of the interventions on online learning engagement.

The future research prospects for online learning engagement are expansive, focusing on using emerging technologies and innovative pedagogical strategies to enhance student engagement. With the development of technologies such as virtual reality, learners can gain interactive and engaging environments, as well as the integration of AI and machine learning to achieve personalized and adaptive learning experiences. Research will also explore the effectiveness of collaborative and peer-assisted learning models in fostering a sense of community and support among online learners. Additionally, studies will investigate the impact of faculty development programs on improving techno-pedagogical skills and instructional design. Overall, these research efforts aim to create more effective, inclusive, and attractive online learning experiences.

## References

[ref1] Abubakari M. S. Nurkhamid N. Priyanto P. (2022). Factors influencing online learning engagement: international students’ perspective and the role of institutional support. Turk. Online J. Dist. Educ. 23, 118–136. doi: 10.17718/tojde.1137253

[ref2] Ahmadi G. Mohammadi A. Asadzandi S. Shah M. Mojtahedzadeh R. (2023). What are the indicators of student engagement in Learning management systems? A systematized review of the literature. Int. Rev. Res. Open Distrib. Learn. 24, 117–136. doi: 10.19173/irrodl.v24i1.6453, PMID: 40009409

[ref3] Appleton J. J. Christenson S. L. Furlong M. (2008). Student engagement with school: critical conceptual and methodological issues of the construct. Psychol. Sch. 45, 369–386. doi: 10.1002/pits.20303

[ref4] Arbaugh J. B. Rau B. L. (2010). A study of disciplinary, structural, and behavioral effects on course outcomes in online MBA courses. Decis. Sci. J. Innov. Educ. 5, 65–95. doi: 10.1111/j.1540-4609.2007.00128.x, PMID: 40035227

[ref5] Artino A. R. Jr. McCoach D. B. (2008). Development and initial validation of the online learning value and self-efficacy scale. J. Educ. Comput. Res. 38, 279–303. doi: 10.2190/EC.38.3.c

[ref6] Astin A. W. (2014). Student involvement: A developmental theory for higher education. In College student development and academic life. eds. Amold K. King I. C. (Routledge), 251–262.

[ref7] Avcı Ü. Ergün E. (2022). Online students’ LMS activities and their effect on engagement, information literacy and academic performance. Interact. Learn. Environ. 30, 71–84. doi: 10.1080/10494820.2019.1636088

[ref8] Bakır-Yalçın E. Usluel Y. K. (2023). Investigating the antecedents of engagement in online learning: do achievement emotions matter? Educ. Inf. Technol. 29, 3759–3791. doi: 10.1007/s10639-023-11995-z, PMID: 40035894

[ref9] Bakker A. B. Demerouti E. (2007). The job demands-resources model: state of the art. J. Manag. Psychol. 22, 309–328. doi: 10.1108/02683940710733115

[ref10] Bandura A. (1977). Self-efficacy: toward a unifying theory of behavioral change. Adv. Behav. Res. Ther. 1, 139–161. doi: 10.1016/0146-6402(78)90002-4, PMID: 847061

[ref11] Barnard L. Lan W. Y. To Y. M. Paton V. O. Lai S.-L. (2009). Measuring self-regulation in online and blended learning environments. Internet High. Educ. 12, 1–6. doi: 10.1016/j.iheduc.2008.10.005

[ref12] Bergdahl N. Nouri J. Fors U. (2020). Disengagement, engagement and digital skills in technology-enhanced learning. Educ. Inf. Technol. 25, 957–983. doi: 10.1007/s10639-019-09998-w

[ref13] Biggs J. (1993). What do inventories of students' learning processes really measure? A theoretical review and clarification. Br. J. Educ. Psychol. 63, 3–19. doi: 10.1111/j.2044-8279.1993.tb01038.x, PMID: 8466833

[ref14] Black A. E. Deci E. L. (2000). The effects of instructors' autonomy support and students' autonomous motivation on learning organic chemistry: A self-determination theory perspective. Sci. Educ. 84, 740–756. doi: 10.1002/1098-237X(200011)84:6<740::AID-SCE4>3.0.CO;2-3

[ref15] Bond M. (2020). Facilitating student engagement through the flipped learning approach in K-12: A systematic review. Comput. Educ. 151:103819. doi: 10.1016/j.compedu.2020.103819

[ref16] Bronfenbrenner U. (1986). Ecology of the family as a context for human development: research perspectives. Dev. Psychol. 22, 723–742. doi: 10.1037/0012-1649.22.6.723

[ref17] Bryson C. Hand L. (2007). The role of engagement in inspiring teaching and learning. Innov. Educ. Teach. Int. 44, 349–362. doi: 10.1080/14703290701602748

[ref18] Buil I. Catalán S. Martínez E. (2020). Engagement in business simulation games: A self-system model of motivational development. Br. J. Educ. Technol. 51, 297–311. doi: 10.1111/bjet.12762

[ref19] Caspari-Sadeghi S. (2022). Applying learning analytics in online environments: measuring learners’ engagement unobtrusively. Front. Educ. 7:840947. doi: 10.3389/feduc.2022.840947, PMID: 40041873

[ref20] Chan S. Lin C. Chau P. Takemura N. Fung J. (2021). Evaluating online learning engagement of nursing students. Nurse Educ Today 104:104985. doi: 10.1016/j.nedt.2021.104985, PMID: 34058645

[ref21] Chen L. (2023). Transactional distance and college students’ Learning engagement in online Learning: the chain mediating role of social presence and autonomous motivation. Psychol. Res. Behav. Manag. 16, 2085–2101. doi: 10.2147/PRBM.S409294, PMID: 37309511 PMC10257921

[ref22] Chen C. M. Li M. C. Liao C. K. (2022). Developing a collaborative writing system with visualization interaction network analysis to facilitate online learning performance. Interact. Learn. Environ. 31, 6054–6073. doi: 10.1080/10494820.2022.2028851

[ref23] Chen T. Peng L. Jing B. Wu C. Yang J. Cong G. J. S. (2020a). The impact of the COVID-19 pandemic on user experience with online education platforms in China. Sustainability 12:7329. doi: 10.3390/su12187329, PMID: 39857420

[ref24] Chen T. Peng L. Yin X. Rong J. Yang J. Cong G. (2020b). Analysis of user satisfaction with online education platforms in China during the COVID-19 pandemic. Healthcare 8:200. doi: 10.3390/healthcare8030200, PMID: 32645911 PMC7551570

[ref25] Cheng K. H. Tsai C. C. (2011). An investigation of Taiwan university students' perceptions of online academic help seeking, and their web-based learning self-efficacy. Internet Higher Educ. 14, 150–157. doi: 10.1016/j.iheduc.2011.04.002

[ref26] Chiu T. K. (2021). Student engagement in K-12 online learning amid COVID-19: A qualitative approach from a self-determination theory perspective. Interact. Learn. Environ. 31, 3326–3339. doi: 10.1080/10494820.2021.1926289

[ref27] Chiu T. K. (2022). Applying the self-determination theory (SDT) to explain student engagement in online learning during the COVID-19 pandemic. J. Res. Technol. Educ. 54, S14–S30. doi: 10.1080/15391523.2021.1891998, PMID: 40036276

[ref28] Cho M. H. Kim Y. Choi D. H. (2017). The effect of self-regulated learning on college students' perceptions of community of inquiry and affective outcomes in online learning. Internet Higher Educ. 34, 10–17. doi: 10.1016/j.iheduc.2017.04.001, PMID: 40035894

[ref29] Coates H. (2010). Development of the Australasian survey of student engagement (AUSSE). High. Educ. 60, 1–17. doi: 10.1007/s10734-009-9281-2

[ref30] Cobb S. C. (2009). Social presence and online learning: a current view from a research perspective. J. Interact. Online Learn. 8, 241–254.

[ref31] Coelho V. Cadima J. Pinto A. I. Guimarães C. (2019). Self-regulation, engagement, and developmental functioning in preschool-aged children. J. Early Interv. 41, 105–124. doi: 10.1177/1053815118810238

[ref32] d’Orville H. J. P. (2020). COVID-19 causes unprecedented educational disruption: Is there a road towards a new normal? Prospects 49, 11–15. doi: 10.1007/s11125-020-09475-0, PMID: 32836420 PMC7268589

[ref33] Davis F. D. (1989). Perceived usefulness, perceived ease of use, and user acceptance of information technology. MIS Q. 13, 319–340. doi: 10.2307/249008

[ref34] Deci E. L. Ryan R. M. (2013). Intrinsic motivation and self-determination in human behavior. Springer Science & Business Media.

[ref35] Demerouti E. Bakker A. B. Nachreiner F. Schaufeli W. B. (2001). The job demands-resources model of burnout. J. Appl. Psychol. 86, 499–512. doi: 10.1037/0021-9010.86.3.499 11419809

[ref36] Derakhshan A. Fathi J. (2023). Grit and foreign language enjoyment as predictors of EFL learners’ online engagement: the mediating role of online learning self-efficacy. Asia Pac. Educ. Res. 33, 759–769. doi: 10.1007/s40299-023-00745-x

[ref37] Dincer A. Yeşilyurt S. Noels K. A. Vargas Lascano D. I. (2019). Self-determination and classroom engagement of EFL learners: A mixed-methods study of the self-system model of motivational development. SAGE Open 9:2158244019853913. doi: 10.1177/2158244019853913

[ref38] Dixson M. D. (2015). Measuring student engagement in the online course: the online student engagement scale (OSE). Online Learn. 19:n4. doi: 10.24059/olj.v19i4.561

[ref39] Donnelly R. Patrinos H. A. J. P. (2021). Learning loss during COVID-19: An early systematic review. Prospects. 51, 601–609. doi: 10.1007/s11125-021-09582-6 34785823 PMC8579897

[ref40] Doo M. Y. Bonk C. J. Shin C. H. Woo B. D. (2020). Structural relationships among self-regulation, transactional distance, and learning engagement in a large university class using flipped learning. Asia Pacific J. Educ. 41, 1–17. doi: 10.1080/02188791.2020.1832020

[ref41] El-Sayad G. Md Saad N. H. Thurasamy R. (2021). How higher education students in Egypt perceived online learning engagement and satisfaction during the COVID-19 pandemic. J. Comput. Educ. 8, 527–550. doi: 10.1007/s40692-021-00191-y

[ref42] Elshami W. Taha M. H. Abdalla M. E. Abuzaid M. Saravanan C. Al Kawas S. (2022). Factors that affect student engagement in online learning in health professions education. Nurse Educ. Today 110:105261. doi: 10.1016/j.nedt.2021.105261, PMID: 35152148

[ref43] Fan S. Chen L. Nair M. Garg S. Yeom S. Kregor G.. (2021). Revealing impact factors on student engagement: learning analytics adoption in online and blended courses in higher education. Educ. Sci. 11:608. doi: 10.3390/educsci11100608

[ref44] Feng L. He L. Ding J. (2023). The association between perceived teacher support, students’ ICT self-efficacy, and online English academic engagement in the blended learning context. Sustain. For. 15:6839. doi: 10.3390/su15086839

[ref45] Findlater G. S. Kristmundsdottir F. Parson S. H. Gillingwater T. H. (2012). Development of a supported self-directed learning approach for anatomy education. Anat. Sci. Educ. 5, 114–121. doi: 10.1002/ase.1255, PMID: 22223487

[ref46] Fosnacht K. (2020). Information literacy’s influence on undergraduates’ learning and development: results from a large multi-institutional study. College and Research Libraries. 81:272. doi: 10.5860/crl.81.2.272

[ref47] Fredricks J. A. Blumenfeld P. C. Paris A. H. (2004). School engagement: potential of the concept, state of the evidence. Rev. Educ. Res. 74, 59–109. doi: 10.3102/00346543074001059, PMID: 38293548

[ref48] Galea S. Merchant R. M. Lurie N. (2020). The mental health consequences of COVID-19 and physical distancing. JAMA Intern Med 180, 817–818. doi: 10.1001/jamainternmed.2020.1562, PMID: 32275292

[ref49] Garrison D. (1997). Self-directed learning: toward a comprehensive model. Adult Educ. Q. 48, 18–33. doi: 10.1177/074171369704800103

[ref50] Garrison D. R. (2016). E-learning in the 21st century: A community of inquiry framework for research and practice. Routledge.

[ref51] Gherghel C. Yasuda S. Kita Y. J. C. (2023). Interaction during online classes fosters engagement with learning and self-directed study both in the first and second years of the COVID-19 pandemic. Comput. Educ. 200:104795. doi: 10.1016/j.compedu.2023.104795, PMID: 37063109 PMC10088368

[ref52] Gross J. J. (1998). Antecedent-and response-focused emotion regulation: divergent consequences for experience, expression, and physiology. J. Pers. Soc. Psychol. 74, 224–237. doi: 10.1037/0022-3514.74.1.224, PMID: 9457784

[ref53] Guo L. Du J. Zheng Q. (2023). Understanding the evolution of cognitive engagement with interaction levels in online learning environments: insights from learning analytics and epistemic network analysis. J. Comput. Assist. Learn. 39, 984–1001. doi: 10.1111/jcal.12781

[ref54] Guo P. J. Kim J. Rubin R. (2014). How video production affects student engagement. Proceedings of the first ACM conference on Learning@ scale conference. 41–50. doi: 10.1145/2556325.2566239

[ref55] Hazzam J. Wilkins S. (2023). The influences of lecturer charismatic leadership and technology use on student online engagement, learning performance, and satisfaction. Comput. Educ. 200:104809. doi: 10.1016/j.compedu.2023.104809

[ref56] Heo H. Bonk C. J. Doo M. Y. (2021). Enhancing learning engagement during COVID-19 pandemic: self-efficacy in time management, technology use, and online learning environments. J. Comput. Assist. Learn. 37, 1640–1652. doi: 10.1111/jcal.12603

[ref57] Hew K. F. (2016). Promoting engagement in online courses: what strategies can we learn from three highly rated MOOCS. Br. J. Educ. Technol. 47, 320–341. doi: 10.1111/bjet.12235

[ref58] Hoi V. N. Le Hang H. J. J. (2021). The structure of student engagement in online learning: a bi-factor exploratory structural equation modelling approach. J. Comput. Assisted Learn. 37, 1141–1153. doi: 10.1111/jcal.12551, PMID: 40035227

[ref59] House R. J. (1976). A 1976 theory of charismatic leadership. Leadership: The cutting edge/Southern Illinois University Press.

[ref60] Hsu H.-C. K. Wang C. V. Levesque-Bristol C. (2019). Reexamining the impact of self-determination theory on learning outcomes in the online learning environment. Educ. Inf. Technol. 24, 2159–2174. doi: 10.1007/s10639-019-09863-w

[ref61] Huang R. Liu D. Guo J. Yang J. Zhao J. Wei X.. (2020). Guidance on flexible learning during campus closures: ensuring course quality of higher education in COVID-19 outbreak. Beijing: Smart Learning Institute of Beijing Normal University.

[ref62] Huang C. Tu Y. He T. Han Z. Wu X. (2024). Longitudinal exploration of online learning burnout: the role of social support and cognitive engagement. Eur. J. Psychol. Educ. 39, 361–388. doi: 10.1007/s10212-023-00693-6 PMC10025060 40479015

[ref63] Johar N. A. Kew S. N. Tasir Z. Koh E. J. S. (2023). Learning analytics on student engagement to enhance students’ learning performance: a systematic review. Sustainability 15:7849. doi: 10.3390/su15107849

[ref64] Jung Y. Lee J. (2018). Learning engagement and persistence in massive open online courses (MOOCS). Comput. Educ. 122, 9–22. doi: 10.1016/j.compedu.2018.02.013

[ref65] Karimah S. N. Hasegawa S. (2022). Automatic engagement estimation in smart education/learning settings: a systematic review of engagement definitions, datasets, and methods. Smart Learn. Environ. 9, 1–48. doi: 10.1186/s40561-022-00212-y PMC9660128 40477985

[ref66] Kicken W. Brand-Gruwel S. Merrinboer J. V. Slot W. (2009). Design and evaluation of a development portfolio: how to improve students' self-directed learning skills. Instr. Sci. 37, 453–473. doi: 10.1007/s11251-008-9058-5

[ref67] Kizilcec R. F. Bailenson J. N. Gomez C. J. (2015). The instructor’s face in video instruction: evidence from two large-scale field studies. J. Educ. Psychol. 107, 724–739. doi: 10.1037/edu0000013

[ref68] Korving H. Hernandez M. De Groot E. (2016). Look at me and pay attention! A study on the relation between visibility and attention in weblectures. Comput. Educ. 94, 151–161. doi: 10.1016/j.compedu.2015.11.011

[ref69] Kuh G. D. (2009). What student affairs professionals need to know about student engagement. J. Coll. Student Dev. 50, 683–706. doi: 10.1353/csd.0.0099

[ref70] Kuh G. D. (2010). The national survey of student engagement: conceptual and empirical foundations. New Dir. Inst. Res. 2009, 5–20. doi: 10.1002/ir.283

[ref71] Kuzu A. Gunuc S. (2015). Confirmation of campus-class-technology model in student engagement: a path analysis. Comput. Hum. Behav. 48, 114–125. doi: 10.1016/j.chb.2015.01.041

[ref72] Lackmann S. Léger P.-M. Charland P. Aubé C. Talbot J. (2021). The influence of video format on engagement and performance in online learning. Brain Sci. 11:128. doi: 10.3390/brainsci11020128, PMID: 33498205 PMC7908978

[ref73] Lai C.-H. Lin H.-W. Lin R.-M. Tho P. D. (2019). Effect of peer interaction among online learning community on learning engagement and achievement. Int. J. Dis. Educ. Technol. 17, 66–77. doi: 10.4018/IJDET.2019010105

[ref74] Li Q. Jiang Q. Liang J.-C. Pan X. Zhao W. (2022). The influence of teaching motivations on student engagement in an online learning environment in China. Australas. J. Educ. Technol. 38, 1–20. doi: 10.14742/ajet.7280

[ref75] Li H. Zhu S. Wu D. Yang H. H. Guo Q. (2023). Impact of information literacy, self-directed learning skills, and academic emotions on high school students’ online learning engagement: a structural equation modeling analysis. Educ. Inf. Technol. 28, 13485–13504. doi: 10.1007/s10639-023-11760-2, PMID: 37361741 PMC10061396

[ref76] Lietaert S. Roorda D. Laevers F. Verschueren K. De Fraine B. (2015). The gender gap in student engagement: the role of teachers’ autonomy support, structure, and involvement. Br. J. Educ. Psychol. 85, 498–518. doi: 10.1111/bjep.12095, PMID: 26446905

[ref77] Liu R.-D. Zhen R. Ding Y. Liu Y. Wang J. Jiang R.. (2018). Teacher support and math engagement: roles of academic self-efficacy and positive emotions. Educ. Psychol. 38, 3–16. doi: 10.1080/01443410.2017.1359238

[ref78] Luan L. Hong J.-C. Cao M. Dong Y. Hou X. J. I. L. E. (2023). Exploring the role of online EFL learners’ perceived social support in their learning engagement: A structural equation model 31, 1703–1714. doi: 10.1080/10494820.2020.1855211, PMID: 40036276

[ref79] Ma Y. Zuo M. Yan Y. Wang K. Luo H. (2022). How do K–12 students’ perceptions of online learning environments affect their online learning engagement? Evidence from China’s COVID-19 school closure period. Sustain. For. 14:15691. doi: 10.3390/su142315691

[ref80] Mailizar M. Abdulsalam A. Suci S. (2020). Secondary school mathematics teachers' views on e-learning implementation barriers during the COVID-19 pandemic: the case of Indonesia. EURASIA J. Math. Sci. Technol. Educ. 16, 1–9. doi: 10.29333/ejmste/8240, PMID: 40041783

[ref81] Martin F. Borup J. (2022). Online learner engagement: conceptual definitions, research themes, and supportive practices. Educ. Psychol. 57, 162–177. doi: 10.1080/00461520.2022.2089147

[ref82] Mayer R. E. (2005). Cognitive theory of multimedia learning. Cambridge Handb. Multimedia Learn. 41, 31–48. doi: 10.1017/CBO9780511816819.004, PMID: 40028382

[ref83] Miao J. Ma L. (2022). Students’ online interaction, self-regulation, and learning engagement in higher education: the importance of social presence to online learning. Front. Psychol. 13:815220. doi: 10.3389/fpsyg.2022.815220, PMID: 36312116 PMC9613360

[ref84] Miner J. B. (2005). Organizational behavior one. Essential theories of motivation and leadership. New York: M.E. Sharpe

[ref85] Moore M. G. (1993). Three types of interaction. Dis. Educ. New Perspect. 3, 1–7. doi: 10.1080/08923648909526659

[ref86] Naragon-Gainey K. McMahon T. P. Chacko T. P. (2017). The structure of common emotion regulation strategies: a meta-analytic examination. Psychol. Bull. 143, 384–427. doi: 10.1037/bul0000093, PMID: 28301202

[ref87] Neuwirth L. S. Jović S. Mukherji B. R. (2021). Reimagining higher education during and post-COVID-19. Challenges Opportunities 27, 141–156. doi: 10.1177/1477971420947738, PMID: 40041252

[ref88] O’Shea S. Stone C. Delahunty J. (2014). "I 'feel' like I am at university even though I am online." exploring how students narrate their engagement with higher education institutions in an online learning environment. Distance Educ. 36, 41–58. doi: 10.1080/01587919.2015.1019970

[ref89] Piccoli G. Ives A. B. (2001). Web-based virtual learning environments: a research framework and a preliminary assessment of effectiveness in basic IT skills training. MIS Q. 25, 401–426. doi: 10.2307/3250989

[ref90] Prakasha S. G. Pramod Kumar M. P. M. Srilakshmi R. (2023). Student engagement in online learning during COVID-19. J. E-Learn. Knowledge Soc. 19, 1–12. doi: 10.20368/1971-8829/1135500

[ref91] Queen C. (2022). Applying charismatic leadership to support learner engagement in virtual environments: teaching and learning in a time of crisis. Pedagogy Health Prom. 9, 158–160.

[ref92] Rashid S. Yadav S. S. (2020). Impact of COVID-19 pandemic on higher education and research. Indian J. Hum. Dev. 14, 340–343. doi: 10.1177/0973703020946700

[ref93] Redmond P. Abawi L. Brown A. Henderson R. Heffernan A. J. (2018). An online engagement framework for higher education 22, 183–204. doi: 10.24059/olj.v22i1.1175, PMID: 33692645

[ref94] Reschly A. L. Christenson S. L. (2012). Jingle, jangle, and conceptual haziness: evolution and future directions of the engagement construct. Springer.

[ref95] Riegel K. Evans T. (2021). Student achievement emotions: examining the role of frequent online assessment. Australas. J. Educ. Technol. 37, 75–87. doi: 10.14742/ajet.6516

[ref96] Rita K. (2011). The challenges to connectivist learning on open online networks: learning experiences during a massive open online course. Int. Rev. Res. Open Dis. Learn. 12, 19–38. doi: 10.19173/irrodl.v12i3.882

[ref97] Roffeei S. H. M. Abdullah N. Basar S. K. R. (2015). Seeking social support on facebook for children with autism spectrum disorders (ASDs). Int. J. Med. Inform. 84, 375–385. doi: 10.1016/j.ijmedinf.2015.01.015, PMID: 25701266

[ref98] Rosli M. S. Saleh N. S. Md. Ali A. Abu Bakar S. (2022). Self-determination theory and online learning in university: advancements, future direction and research gaps. Sustainability 14:14655. doi: 10.3390/su142114655, PMID: 39857420

[ref99] Ryan R. M. Deci E. L. (2017). Self-determination theory: basic psychological needs in motivation, development, and wellness. Guilford Publications.

[ref100] Ryan R. M. Deci E. L. (2020). Intrinsic and extrinsic motivation from a self-determination theory perspective: definitions, theory, practices, and future directions. Contemp. Educ. Psychol. 61:101860. doi: 10.1016/j.cedpsych.2020.101860

[ref101] Sahu P. J. C. (2020). Closure of universities due to coronavirus disease 2019 (COVID-19): impact on education and mental health of students and academic staff. Cureus 12:e7541. doi: 10.7759/cureus.7541, PMID: 32377489 PMC7198094

[ref102] Stefansson K. K. Gestsdottir S. Birgisdottir F. Lerner R. M. (2018). School engagement and intentional self-regulation: a reciprocal relation in adolescence. J. Adolesc. 64, 23–33. doi: 10.1016/j.adolescence.2018.01.005, PMID: 29408096

[ref103] Sun W. Hong J.-C. Dong Y. Huang Y. Fu Q. (2023). Self-directed learning predicts online learning engagement in higher education mediated by perceived value of knowing learning goals. Asia Pac. Educ. Res. 32, 307–316. doi: 10.1007/s40299-022-00653-6

[ref104] Sun J. C. Y. Rueda R. (2012). Situational interest, computer self-efficacy and self-regulation: their impact on student engagement in distance education. Br. J. Educ. Technol. 43, 191–204. doi: 10.1111/j.1467-8535.2010.01157.x

[ref105] Sung E. Mayer R. E. (2012). Five facets of social presence in online distance education. Comput. Hum. Behav. 28, 1738–1747. doi: 10.1016/j.chb.2012.04.014

[ref106] Tsai C.-L. Ku H.-Y. Campbell A. (2021). Impacts of course activities on student perceptions of engagement and learning online. Distance Educ. 42, 106–125. doi: 10.1080/01587919.2020.1869525

[ref107] Vollet J. W. Kindermann T. A. Skinner E. A. (2017). In peer matters, teachers matter: Peer group influences on students’ engagement depend on teacher involvement. J. Educ. Psychol. 109, 635–652. doi: 10.1037/edu0000172

[ref108] Waite M. Mackness J. Roberts G. Lovegrove E. (2013). Liminal participants & skilled orienteers: a case study of learner participation in a MOOC for new lecturers. J Online Learn Teaching. 9, 51–65.

[ref109] Wang R. Cao J. Xu Y. Li Y. (2022). Learning engagement in massive open online courses: a systematic review. Front. Educ. 7:1074435. doi: 10.3389/feduc.2022.1074435

[ref110] Wang M. T. Fredricks J. A. Ye F. Hofkens T. L. Linn J. S. (2016). The math and Science engagement scales: scale development, validation, and psychometric properties. Learn. Instr. 43, 16–26. doi: 10.1016/j.learninstruc.2016.01.008

[ref111] Watts L. (2016). Synchronous and asynchronous communication in distance learning: a review of the literature. Q. Rev. Dis. Educ. 17:23–32.

[ref112] Werang B. R. Leba S. M. R. (2022). Factors affecting student engagement in online teaching and learning: a qualitative case study. Qual. Rep. 27, 555–577. doi: 10.46743/2160-3715/2022.5165, PMID: 39386316

[ref113] Zang F. Tian M. Fan J. Sun Y. (2022). Influences of online learning environment on international students’ intrinsic motivation and engagement in the Chinese learning. J. Int. Stud. 12, 61–82. doi: 10.32674/jis.v12iS1.4608

[ref114] Zheng C. Liang J.-C. Chai C. S. Chen X. Liu H. (2023). Comparing high school students’ online self-regulation and engagement in English language learning. J. Syst. 115:103037. doi: 10.1016/j.system.2023.103037, PMID: 40035894

[ref115] Zhoc K. C. Cai Y. Yeung S. S. Shan J. (2022). Subjective wellbeing and emotion regulation strategies: how are they associated with student engagement in online learning during COVID-19? Br. J. Educ. Psychol. 92, 1537–1549. doi: 10.1111/bjep.12513, PMID: 35567326 PMC9347556

[ref116] Zhou S. Zhu H. Zhou Y. (2022). Impact of teenage EFL learners’ psychological needs on learning engagement and behavioral intention in synchronous online English courses. Sustain. For. 14:10468. doi: 10.3390/su141710468

[ref117] Zimmerman B. J. (2002). Becoming a self-regulated learner: an overview. Theory Pract. 41, 64–70. doi: 10.1207/s15430421tip4102_2

[ref118] Zimmerman W. A. Kulikowich J. M. (2016). Online learning self-efficacy in students with and without online learning experience. Am. J. Dist. Educ. 30, 180–191. doi: 10.1080/08923647.2016.1193801

