# Supplementary material for: What are the influencing factors of online learning engagement? A systematic literature review
Source: Front Psychol. 2025 Mar 17;16:1542652. doi: 10.3389/fpsyg.2025.1542652 (PMC11955628; doi:10.3389/fpsyg.2025.1542652)
Supplement: Supplementary file 1 [file Supplementary_file_1.pdf]

Appendix 1: Selected literatures in this study

| Article | Nation | Types of Learner | Types of Engagement | Source of Data | Samples | Factors |
|---|---|---|---|---|---|---|
| (Zheng et al., 2023) | China | high school | Cognitive<br>Behavioural<br>Emotional<br>Social | questionnaire | 707 | Demography<br>Self-regulation |
| (Luan et al., 2023) | China | university | Cognitive<br>Behavioural<br>Emotional<br>Social | questionnaire | 615 | Perceived social support |
| (Derakhshan & Fathi, 2024) | Iran | university | Cognitive<br>Behavioural<br>Emotional<br>Social | questionnaire | 578 | Grit<br>Foreign language enjoyment<br>Self-efficacy |
| (Li et al., 2023) | China | high school | Cognitive<br>Behavioural<br>Emotional<br>Social | questionnaire | 1993 | Information literacy<br>Self-directed learning skills<br>Academic emotions |
| (Luo et al., 2023) | China | university | Cognitive<br>Behavioural<br>Emotional | questionnaire | 1829 | Instructor Humor<br>Academic emotions |
| (Gherghel et al., 2023) | Japan | university | Behavioural<br>Emotinal | questionnaire | 1167 | Social interaction |
| (Bakır-Yalçın & Usluel, 2024) | Turkey | university | Cognitive<br>Behavioural<br>Emotional | questionnaire | 1450 | Control of Learning Beliefs<br>Task Value<br>Achievement Emotions<br>Emotion Reguraltion Strategy |
| (Munangatire & Indjamba, 2023) | Namibia | university | Cognitive<br>Behavioural<br>Emotional | interview | 12 | Prior knowledge<br>Technology<br>Online learning platforms |
| (Huang et al., 2024) | China | university | Cognitive | questionnaire | 116 | Social support |
| (Jung & Lee, 2018) | Korea | university | Cognitive<br>Behavioural<br>Emotional | questionaire | 306 | Academic self-efficacy<br>Teaching presence |

| | | | | | | |
|---|---|---|---|---|---|---|
| | | | | | | Perceived usefulness<br>Perceived ease of use |
| (Tran-Duong & Vo-Thi) | Vietnam | university | Cognitive<br>Behavioural<br>Emotional<br>Social | questionnaire | 1015 | Social media literacy |
| (Sun et al., 2023) | China | university | Cognitive<br>Behavioural<br>Emotional | questionnaire | 497 | Self-directed Learning Attitude<br>Self-directed Learning Approach<br>Perceived Value of Knowing |
| (Prakasha et al., 2023) | India | university | Cognitive<br>Behavioural<br>Emotional | questionnaire | 600 | Learning Goals<br>Intrinsic motivation<br>Demographic variables |
| (Zapata-Cuervo et al., 2023) | U.S.<br>South Korea<br>Colombia | university | Comprehensive | questionnaire | 523 | Motivation<br>Self-efficacy<br>Anxiety |
| (Feng et al., 2023) | China | university | Cognitive<br>Behavioural<br>Emotional<br>Social | questionnaire | 960 | Perceived teacher support<br>Students' ICT self-efficacy |
| (Dvorakova et al., 2023) | Czech | university | Behavioural | questionnaire | 129 | Technical equipment<br>Physical environment |
| (Hazzam & Wilkins, 2023) | U.S. | university | Cognitive<br>Behavioural<br>Emotional | questionnaire | 659 | Lecturer charismatic leadership skills<br>Lecturer technology use skills |
| (McLeod & Gupta, 2023) | U.S. | university | Comprehensive | interview | 15 | Self-efficacy<br>Psychological safety |

| | | | | | | |
|---|---|---|---|---|---|---|
| (Chen, 2023) | China | university | Comprehensive | questionnaire | 827 | Transactional distance<br>Social Presence<br>Autonomous motivation |
| (Guo et al., 2023) | China | university | cognitive | text data from discussion forums | 1474 | Learners' interactions |
| (Azila-Gbettor et al., 2023) | Ghana | university | Comprehensive | questionnaire | 310 | University support<br>Students' vitality |
| (Chiu, 2022) | China | secondary school | Cognitive<br>Behavioural<br>Emotional<br>Social | questionnaire | 1201 | Motivation |
| (Deng et al., 2022) | China | high school | Cognitive<br>Behavioural<br>Emotional | questionnaire | 926 | Self-efficacy<br>Academic emotion |
| (Wang, 2022) | China | university | Cognitive<br>Behavioural<br>Emotional | questionnaire | 1328 | Teaching presence |
| (Pan, 2022) | China | university | Comprehensive | questionnaire | 392 | Educational situation perception<br>Teacher support<br>Self-efficacy |
| (Werang & Leba, 2022) | Indonesia | university | Comprehensive | interview | 10 | Technological Tools<br>Technology experience<br>Learning habits |
| (Elshami et al., 2022) | United Arab Emirates | university | Comprehensive | questionnaire | 472 | Collaborative Learing<br>Techno-Pedagogical Skills<br>Peer Assisted Learning<br>Self Directed Learning |
| (Ma et al., 2022) | China | high school | Cognitive<br>Behavioural<br>Emotional | questionnaire | 1092 | Online learning environment |

| | | | | | | |
|---|---|---|---|---|---|---|
| (Wickremasinghe & Kumuduni, 2022) | Sri Lanka | university | Cognitive<br>Behavioural<br>Emotional | questionnaire | 238 | Physical environment |
| (Zhou et al., 2022) | China | secondary school | Cognitive<br>Behavioural<br>Emotional<br>Social | questionnaire | 232 | Motivation |
| (Zang et al., 2022) | China | university | Cognitive<br>Behavioural<br>Emotional | questionnaire | 63 | Online Learning Environment<br>Intrinsic Motivation |
| (Y. Wang et al., 2022) | China | university | Comprehensive | questionnaire | 515 | Interactions<br>Self-efficacy<br>Academic emotions |
| (X. Wang et al., 2022) | China | university | Cognitive<br>Behavioural<br>Emotional<br>Social | questionnaire | 408 | Digital Nativity<br>Self-Regulated Learning |
| (Dai et al., 2022) | China | secondary school | Cognitive<br>Behavioural<br>Emotional | questionnaire | 428 | Self-regulated learning<br>Perceived academic control |
| (Miao & Ma, 2022) | China | university | Cognitive<br>Behavioural<br>Emotional | questionnaire | 334 | Social presence<br>Online interaction<br>Self-regulation |
| (Zhoc et al., 2022) | China | university | Cognitive<br>Behavioural<br>Emotional | questionnaire | 965 | students' subjective wellbeing<br>emotion regulation strategies |
| (Miao et al., 2022) | China | university | Comprehensive | questionnaire | 354 | Teacher-Student Interaction<br>Student-Student Interaction<br>Social Presence |
| (Chen et al., 2022) | China | university | Behavioural | questionnaire | 606 | Self-compassion<br>Positive emotion<br>Self-improvement motivation |

| | | | | | | |
|---|---|---|---|---|---|---|
| (Li et al., 2022) | China | university | Cognitive<br>Behavioural<br>Emotional<br>Social | questionnaire | 414 | Teaching motivations<br>Student intrinsic motivation<br>Student extrinsic motivation |
| (Nichter, 2021) | U.S. | university | Behavioural | questionnaire | 1641 | Use of mobile devices |
| (Heo et al., 2021) | Korea | university | Comprehensive | questionnaire | 1205 | self-efficacy |
| (El-Sayad et al., 2021) | Egypt | university | Cognitive<br>Behavioural<br>Emotional | questionnaire | 330 | Academic self-efficacy,<br>Perceived usefulness of online learning systems,<br>teaching presence |
| (Tsai et al., 2021) | U.S | university | Cognitive<br>Behavioural<br>Emotional<br>Social | questionnaire | 371 | Interactions |
| (Kuo et al., 2021) | China | university | Cognitive<br>Behavioural<br>Emotional | questionnaire | 608 | Web-based learning self-efficacy<br>Online academic hardiness |
| (Gamage et al., 2021) | U.K. | university | Comprehensive | questionaire<br>interview | 120<br>20 | Instructor's Coaching and mentoring |
| (Domina et al., 2021) | U.S. | secondary school | Cognitive<br>Behavioural<br>Emotional | questionaire | 1000 | Household material and technological resources<br>School programming and instructional strategies<br>Family social capital |
| (Fan et al., 2021) | Australia | university | Behavioural | log of LMS | 5906 | Teaching Presence:<br>Teacher Input<br>Course Content |

| | | | | | | |
|---|---|---|---|---|---|---|
| (Li et al., 2021) | China | secondary school | Cognitive Behavioural Emotional | interview | 29 | Teacher presence Parental involvement Supportive learning Environment/community |
| (Chiu, 2021) | China | high school | Cognitive Behavioural Emotional | interview | 54 | Perceived of autonomy Perceived of competence Perceived of relatedness |
| (Han et al., 2021) | China | university | Behavioural Emotional | questionaire | 428 | Student cohesiveness Teacher support Student involvement |
| (Erdoğdu & Çakıroğlu, 2021) | Turkey | university | Cognitive Behavioural Emotional | log of LMS interview | 74 | Self-Efficacy Instructor's humor |
| (Alemayehu & Chen, 2021) | China | university | Comprehensive | questionnaire | 354 | Students' motivation Learning self-efficacy Self-monitoring |
| (Lackmann et al., 2021) | Canada | university | Emotional Cognitive | neurophysiological instruments | 26 | Video Format |
| (Xu et al., 2020) | China | university | Cognitive Behavioural Emotional | questionaire | 46 | Teacher facilitation |
| (Abubakari et al., 2022) | Indonesia | university | Cognitive Behavioural Emotional | questionaire | 102 | University support Motivation Personal innovativeness |

Abubakari, M. S., Nurkhamid, N., & Priyanto, P. (2022). FACTORS INFLUENCING ONLINE LEARNING ENGAGEMENT: INTERNATIONAL STUDENTS'PERSPECTIVE AND THE ROLE OF INSTITUTIONAL SUPPORT. *Turkish Online Journal of Distance Education*, *23*(3),

118-136.doi: 10.17718/tojde.1137253

Alemayehu, L., & Chen, H.-L. (2021). The influence of motivation on learning engagement: The mediating role of learning self-efficacy and self-monitoring in online learning environments. *Interactive Learning Environments*, *31*(7), 4605-4618. doi: 10.1080/10494820.2021.1977962

Azila-Gbettor, E. M., Abiemo, M. K., & Glate, S. N. (2023). University support and online learning engagement during the Covid-19 period: The role of student vitality. *Heliyon*, *9*(1), e12832.doi: 10.1016/j.heliyon.2023.e12832

Bakır-Yalçın, E., & Usluel, Y. K. (2024). Investigating the antecedents of engagement in online learning: do achievement emotions matter? *Education and information technologies*, *29*(4), 3759-3791.doi: 10.1007/s10639-023-11995-z

Chen, J., Lin, G., & Lyu, Y. (2022). The impact of self-compassionate mindfulness on online learning behavioral engagement of international students during COVID-19: Positive emotion and self-improvement motivation as mediators. *Frontiers in psychology*, *13*, 969657. doi:10.3389/fpsyg.2022.969657.

Chen, L. (2023). Transactional Distance and College Students' Learning Engagement in Online Learning: The Chain Mediating Role of Social Presence and Autonomous Motivation. *Psychology Research Behavior Management*, *16*, 2085-2101. doi:10.2147/PRBM.S409294.

Chiu, T. K. (2021). Student engagement in K-12 online learning amid COVID-19: A qualitative approach from a self-determination theory perspective. *Interactive Learning Environments*, *31*(6), 3326-3339.doi: 10.1080/10494820.2021.1926289

Chiu, T. K. (2022). Applying the self-determination theory (SDT) to explain student engagement in online learning during the COVID-19 pandemic. *Journal of Research on Technology in Education*, *54*(sup1), S14-S30.doi: 10.1080/15391523.2021.1891998

Dai, W., Li, Z., & Jia, N. (2022). Self-regulated learning, online mathematics learning engagement, and perceived academic control among Chinese junior high school students during the COVID-19 pandemic: A latent profile analysis and mediation analysis. *Frontiers in Psychology*, *13*, 1042843. doi:10.3389/fpsyg.2022.1042843.

Deng, W., Lei, W., Guo, X., Li, X., Ge, W., & Hu, W. (2022). Effects of regulatory focus on online learning engagement of high school students: The mediating role of self - efficacy and academic emotions. *Journal of Computer Assisted Learning*, *38*(3), 707-718.doi: 10.1111/jcal.12642

Derakhshan, A., & Fathi, J. (2024). Grit and foreign language enjoyment as predictors of EFL learners' online engagement: The mediating role of online learning self-efficacy. *The Asia-Pacific Education Researcher*, *33*, 759-769. doi:10.1007/s40299-023-00745-x.

Domina, T., Renzulli, L., Murray, B., Garza, A. N., & Perez, L. (2021). Remote or removed: Predicting successful engagement with online learning during COVID-19. *Socius*, *7*, 2378023120988200.doi: 10.1177/2378023120988200

Dvorakova, K., Emmer, J., Janktova, R., & Klementová, K. (2023). The influence of remote learning environment and use of technology on university students' behavioural engagement in contingency online learning. *Tuning Journal for Higher Education*, *10*(2), 271-300.

El-Sayad, G., Md Saad, N. H., & Thurasamy, R. (2021). How higher education students in Egypt perceived online learning engagement and satisfaction during the COVID-19 pandemic. *Journal of Computers in Education*, *8*(4), 527-550.doi: 10.1007/s40692-021-00191-y

Elshami, W., Taha, M. H., Abdalla, M. E., Abuzaid, M., Saravanan, C., & Al Kawas, S. (2022). Factors that affect student engagement in online learning in health professions education. *Nurse*

*Education Today*, *110*, 105261. doi:10.1016/j.nedt.2021.105261.

Erdoğdu, F., & Çakıroğlu, Ü. (2021). The educational power of humor on student engagement in online learning environments. *Research and Practice in Technology Enhanced Learning*, *16*(1), 1-25.doi: 10.1186/s41039-021-00158-8

Fan, S., Chen, L., Nair, M., Garg, S., Yeom, S., Kregor, G.,…Wang, Y. (2021). Revealing impact factors on student engagement: Learning analytics adoption in online and blended courses in higher education. *Education Sciences*, *11*(10), 608. doi:10.3390/educsci11100608.

Feng, L., He, L., & Ding, J. (2023). The Association between Perceived Teacher Support, Students' ICT Self-Efficacy, and Online English Academic Engagement in the Blended Learning Context. *Sustainability*, *15*(8), 6839. doi:10.3390/su15086839.

Gamage, K. A., Perera, D. S., & Wijewardena, M. D. N. (2021). Mentoring and coaching as a learning technique in higher education: The impact of learning context on student engagement in online learning. *Education Sciences*, *11*(10), 574. doi:10.3390/educsci11100574.

Gherghel, C., Yasuda, S., & Kita, Y. (2023). Interaction during online classes fosters engagement with learning and self-directed study both in the first and second years of the COVID-19 pandemic. *Computers & Education*, *200*, 104795. doi:10.1016/j.compedu.2023.104795.

Guo, L., Du, J., & Zheng, Q. (2023). Understanding the evolution of cognitive engagement with interaction levels in online learning environments: Insights from learning analytics and epistemic network analysis. *Journal of Computer Assisted Learning*, *39*(3), 984-1001.doi: 10.1111/jcal.12781

Han, J., Geng, X., & Wang, Q. (2021). Sustainable development of university EFL learners' engagement, satisfaction, and self-efficacy in online learning environments: Chinese experiences. *Sustainability*, *13*(21), 11655. doi:10.3390/su132111655.

Hazzam, J., & Wilkins, S. (2023). The influences of lecturer charismatic leadership and technology use on student online engagement, learning performance, and satisfaction. *Computers & Education*, *200*, 104809. doi:10.1016/j.compedu.2023.104809.

Heo, H., Bonk, C. J., & Doo, M. Y. (2021). Enhancing learning engagement during COVID‐19 pandemic: Self‐efficacy in time management, technology use, and online learning environments. *Journal of Computer Assisted Learning*, *37*(6), 1640-1652.doi: 10.1111/jcal.12603

Huang, C., Tu, Y., He, T., Han, Z., & Wu, X. (2024). Longitudinal exploration of online learning burnout: the role of social support and cognitive engagement. *European Journal of Psychology of Education*, *39*(1), 361-388.doi: 10.1007/s10212-023-00693-6

Jung, Y., & Lee, J. (2018). Learning engagement and persistence in massive open online courses (MOOCS). *Computers & Education*, *122*, 9-22. doi:10.1016/j.compedu.2018.02.013.

Kuo, T. M., Tsai, C.-C., & Wang, J.-C. (2021). Linking web-based learning self-efficacy and learning engagement in MOOCs: The role of online academic hardiness. *The Internet and Higher Education*, *51*, 100819. doi:10.1016/j.iheduc.2021.100819.

Lackmann, S., Léger, P.-M., Charland, P., Aubé, C., & Talbot, J. (2021). The influence of video format on engagement and performance in online learning. *Brain Sciences*, *11*(2), 128. doi:10.3390/brainsci11020128.

Li, F., Jin, T., Edirisingha, P., & Zhang, X. (2021). School-aged students' sustainable online learning engagement during covid-19: community of inquiry in a chinese secondary education context. *Sustainability*, *13*(18), 10147. doi:10.3390/su131810147.

Li, H., Zhu, S., Wu, D., Yang, H. H., & Guo, Q. (2023). Impact of information literacy, self-directed learning skills, and academic emotions on high school students' online learning engagement: A structural equation modeling analysis. *Education and information technologies*, *28*, 13485–13504 doi:10.1007/s10639-023-11760-2.

Li, Q., Jiang, Q., Liang, J.-C., Pan, X., & Zhao, W. (2022). The influence of teaching motivations on student engagement in an online learning environment in China. *Australasian Journal of Educational Technology*, *38*(6), 1-20.doi: 10.14742/ajet.7280

Luan, L., Hong, J.-C., Cao, M., Dong, Y., & Hou, X. (2023). Exploring the role of online EFL learners' perceived social support in their learning engagement: A structural equation model. *Interactive Learning Environments*, *31*(3), 1703-1714.doi: 10.1080/10494820.2020.1855211

Luo, R., Zhan, Q., & Lyu, C. J. (2023). Influence of instructor humor on learning engagement in the online learning environment. *Social Behavior Personality: an international journal*, *51*(2), 1-12.doi: 10.2224/sbp.12145

Ma, Y., Zuo, M., Yan, Y., Wang, K., & Luo, H. (2022). How Do K–12 Students' Perceptions of Online Learning Environments Affect Their Online Learning Engagement? Evidence from China's COVID-19 School Closure Period. *Sustainability*, *14*(23), 15691. doi:10.3390/su142315691.

McLeod, E., & Gupta, S. J. M. s. e. (2023). The role of psychological safety in enhancing medical students' engagement in online synchronous learning. *Medical science educator*, *33*(2), 423-430.doi: 10.1007/s40670-023-01753-8

Miao, J., Chang, J., & Ma, L. (2022). Teacher–student interaction, student–student interaction and social presence: their impacts on learning engagement in online learning environments. *The Journal of Genetic Psychology*, *183*(6), 514-526.doi: 10.1080/00221325.2022.2094211

Miao, J., & Ma, L. (2022). Students' online interaction, self-regulation, and learning engagement in higher education: The importance of social presence to online learning. *Frontiers in Psychology*, *13*, 815220. doi:10.3389/fpsyg.2022.815220.

Munangatire, T., & Indjamba, L. (2023). Learning engagement; nursing students' experiences in an online environment at a university. *Nursing Open*, *10*(5), 3145-3152.doi: 10.1002/nop2.1564

Nichter, S. (2021). Does mode of access make a difference? Mobile learning and online student engagement. *Online learning*, *25*(3), 5-17.doi: 10.24059/olj.v25i3.2848

Pan, X. (2022). Exploring the multidimensional relationships between educational situation perception, teacher support, online learning engagement, and academic self-efficacy in technology-based language learning. *Frontiers in Psychology*, *13*, 1000069. doi:10.3389/fpsyg.2022.1000069.

Prakasha, S., MPM, P. K., & Srilakshmi, R. (2023). Student engagement in online learning during COVID-19. *Journal of e-Learning Knowledge Society*, *19*(1), 1-12.doi: 10.20368/1971-8829/1135500

Sun, W., Hong, J.-C., Dong, Y., Huang, Y., & Fu, Q. (2023). Self-directed learning predicts online learning engagement in higher education mediated by perceived value of knowing learning goals. *The Asia-Pacific Education Researcher*, *32*(3), 307-316.doi: 10.1007/s40299-022-00653-6

Tran‐Duong, Q. H., & Vo‐Thi, N. T. (2023). The influence of social media literacy on student engagement in online learning. *Journal of Computer Assisted Learning*, *39*(6), 1888-1901.doi: 10.1111/jcal.12849

Tsai, C.-L., Ku, H.-Y., & Campbell, A. (2021). Impacts of course activities on student perceptions of engagement and learning online. *Distance education*, *42*(1), 106-125.doi:

10.1080/01587919.2020.1869525

Wang, X., Hui, L., Jiang, X., & Chen, Y. (2022). Online English learning engagement among digital natives: the mediating role of self-regulation. *Sustainability*, *14*(23), 15661. doi:10.3390/su142315661.

Wang, Y. (2022). Effects of teaching presence on learning engagement in online courses. *Distance Education*, *43*(1), 139-156.doi: 10.1080/01587919.2022.2029350

Wang, Y., Cao, Y., Gong, S., Wang, Z., Li, N., & Ai, L. (2022). Interaction and learning engagement in online learning: The mediating roles of online learning self-efficacy and academic emotions. *Learning and Individual Differences*, *94*, 102128. doi:10.1016/j.lindif.2022.102128.

Werang, B. R., & Leba, S. M. R. (2022). Factors Affecting Student Engagement in Online Teaching and Learning: A Qualitative Case Study. *Qualitative Report*, *27*(2), 555-577.doi: 10.46743/2160-3715/2022.5165

Wickremasinghe, H. T., & Kumuduni, W. (2022). Impact of Physical Learning Environment on University Students' Academic Engagement in an Online Learning Setting during Covid-19: Evidence from a Sri Lankan University. *International Journal of Built Environment and Sustainability*, *9*(3), 35-46.doi: 10.11113/ijbes.v9.n3.953

Xu, B., Chen, N.-S., & Chen, G. (2020). Effects of teacher role on student engagement in WeChat-Based online discussion learning. *Computers & Education*, *157*, 103956. doi:10.1016/j.compedu.2020.103956.

Zang, F., Tian, M., Fan, J., & Sun, Y. (2022). Influences of online learning environment on international students' intrinsic motivation and engagement in the Chinese learning. *Journal of International Students*, *12*(S1), 61-82.doi: 10.32674/jis.v12iS1.4608

Zapata-Cuervo, N., Montes-Guerra, M. I., Shin, H. H., Jeong, M., & Cho, M.-H. (2023). Students' psychological perceptions toward online learning engagement and outcomes during the COVID-19 pandemic: A comparative analysis of students in three different countries. *Journal of Hospitality & Tourism Education*, *35*(2), 108-122.doi: 10.1080/10963758.2021.1907195

Zheng, C., Liang, J.-C., Chai, C. S., Chen, X., & Liu, H. (2023). Comparing high school students' online self-regulation and engagement in English language learning. *System*, *115*, 103037. doi:10.1016/j.system.2023.103037.

Zhoc, K. C., Cai, Y., Yeung, S. S., & Shan, J. (2022). Subjective wellbeing and emotion regulation strategies: How are they associated with student engagement in online learning during Covid‐19? *British Journal of Educational Psychology*, *92*(4), 1537-1549.doi: 10.1111/bjep.12513

Zhou, S., Zhu, H., & Zhou, Y. (2022). Impact of teenage EFL learners' psychological needs on learning engagement and behavioral intention in synchronous online English courses. *Sustainability*, *14*(17), 10468. doi:10.3390/su141710468.
